# Psychotropic drug use rate among detention house residents and association with the category of the crimes in Japan

**DOI:** 10.1002/npr2.12203

**Published:** 2021-08-25

**Authors:** Akihiro Nishio

**Affiliations:** ^1^ Health Administration Center Gifu University Gifu Japan

**Keywords:** crimes, detention houses, Japan, mental illness, psychotropic drugs

## Abstract

**Aim:**

The White Paper on Crime 2019 from the Japanese Ministry of Justice reported that the percentage of crimes committed by people with mental disabilities was only 1.0%. In contrast, the findings of a statistical survey of correctional facilities reported that 15.1% of the prisoners were diagnosed with a mental illness. This study aimed at clarifying the relationship between mental illness and crime among suspects in a detention house and explaining this large gap.

**Methods:**

Criminal suspects who were newly admitted in the Gifu detention house in Japan were eligible for the study. The status of psychotropic drug use was investigated, and its relationship with age, sex, offense history, and type of crime was analyzed. Newly prescribed medications in detention houses or police stations were excluded.

**Results:**

In total, 26.5% of the residents in a detention house used psychotropic drugs. The psychotropic drug use rate was 16.7% (excluding the sleeping pill use rate). The use rates of sleeping pills, anxiolytics, antidepressants, and antipsychotics were 22.6%, 11.1%, 3.0%, and 9.6%, respectively. Psychotropic drug use was high in illicit drug users and low in suspects for immigration violence. Psychotropic drug use was higher among female suspects, suspects in their 40s and 50s, and suspects with a multiple crime history. Anxiolytic (17.0%) and antipsychotic (11.9%) use rates were high among suspects for violence.

**Conclusion:**

In total, 26.5% of the subjects used psychotropic drugs. Psychotropic drug use was high in illicit drug users and low in suspects for immigration violence.

## Abstract 2

In total, 26.5% of the residents in a detention house used psychotropic drugs. The psychotropic drug use rate was 16.7% (excluding the sleeping pill use rate). The use rates of sleeping pills, anxiolytics, antidepressants, and antipsychotics were 22.6%, 11.1%, 3.0%, and 9.6%, respectively.

## Graphical abstract

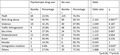

## 1 INTRODUCTION

In Western countries, mental illnesses are related to all or some crime categories, such as arson and violence.^1^, ^2^, ^3^, ^4^, ^5^, ^6^, ^7^, ^8^ However, the White Paper on Crime, 2019, from the Japanese Ministry of Justice reported that the percentage of crimes committed by people with mental disabilities was only 1.0%.^9^ The Cabinet Office of Japan reported that the percentage of people with mental illnesses nationwide was 3.3%^10^; thus, it was concluded that people with mental illnesses were less likely to commit crimes than healthy people. In contrast, the Japanese Ministry of Justice published the findings of a statistical survey of correctional facilities, which reported that 15.1% of the prisoners were diagnosed with a mental illness.^11^ In a bid to understand this large discrepancy, several questions have been asked; these include the following questions: How can this large gap be explained? Are prisoners more susceptible to mental illnesses during their imprisonment? Are mental illnesses simply neglected in the judgment process?

There are no reports or surveys on the relationship between mental illness and crime in Japan, except for the statistical survey of correctional facilities mentioned above. This survey targeted only new prisoners and reported that the rates of intellectual disability, personality disorders, neurotic disorders, others, and unknown were 1.5%, 0.4%, 2.4%, 10.5%, and 0.3%, respectively. Since the largest group was categorized just like others, the real‐world situation could not be deduced from this survey. People who were diagnosed with a mental illness after their arrest might have been included in the survey. This may have affected the nature of the crime, judgment process, and prolonged imprisonment duration in the detention house. Thus, the pure relationship between mental illnesses and crime could not be assessed using these data.

In other countries than Japan, some studies have reported the rate of mental illnesses among prisoners. Fazel et al^12^ conducted a systematic review of mental disorders among prisoners in Western countries in 2001 and reported that 3%–7% of men had psychotic illnesses; of these, 10% had major depression and 65% had personality disorders, with 47% having antisocial personality disorders. They also reported that 4% of women had psychotic illnesses; of these, 12% had major depression and 42% had a personality disorder, with 21% having antisocial personality disorder. Seth et al^13^ conducted a similar systematic review in the United States and reported that the prevalence of any mental illness was 10%–31%, excluding substance abuse. In Ireland, Gulati et al^14^ reported that the percentage of patients with psychotic disorders was 3.6%, that of patients with affective disorders was 4.3%, that of patients with alcohol use disorder was 28.3%, and that of patients with substance use disorder was 50.9%. In Asian countries, Tung TH^15^ reported a prevalence of 11.31% of mental illness in Taiwanese prisoners. In Hong Kong, Chow et al^16^ reported that 39.6% (46% men and 29.5% women) of the prisoners had a current psychiatric disorder. All the studies reported that the prevalence of mental illnesses in prisoners was much higher than that in the general population. The relationship between mental illness and crime could not be determined from these studies because the targeted subjects were already imprisoned. Thus, its influence on imprisonment or the court's judgment could not be avoided.

Although our original research question was “what percentage of criminals are considered to have a mental illness at the time of arrest?,” we changed our research question to “what percentage of suspects in the detention house used psychotropic drugs at the time of arrest?” The reason we focused on psychotropic drug use was that it was extremely difficult to obtain diagnostic information from criminals or suspects in Japan. In contrast, we could easily obtain information of suspect's past psychotropic drug use in a detention house, which provided following benefit. In Japan, prisoners are sent to different types of prisons according to their crime and past history of the crime. Therefore, even if we obtained the data from several prisons in Japan, those data would not represent the general demographic of criminals. Detention houses admit all suspects in the region who committed the crime and are awaiting the court's final judgment, of whom 99% are usually charged guilty. Thus, the data of suspects in the detention house somehow represent the general demographics of criminals in Japan. Furthermore, we analyzed the relationship between psychotropic drug use and the crime category. To the best of our knowledge, no similar surveys have been conducted in Japan or other countries. This study may bring new perspectives on the relationship between crime and mental illness and a new strategy to decrease crimes committed by people with mental illness.

## 2 METHODS

### 2.1 Subjects

Criminal suspects who were newly admitted in the Gifu detention house in Japan from December 1, 2017, to November 30, 2019 were eligible for the study. The Gifu detention house is located in Gifu City, which is the capital of Gifu Prefecture. This is a typical middle‐sized city in Japan. The medical staff of the detention house gathered the suspects’ information, including sex, age, history of medication use, type of crime, and other information. We collected the data after anonymization. The status of psychotropic drug use was investigated and confirmed by referring to hospitals sometimes, and its relationship with age, sex, offense history, and type of crime was analyzed. Newly prescribed medications in detention houses or police stations were excluded. Medications are free in the detention house.

### 2.2 Categorization of crimes and psychotropic drugs

The crime categories included theft, illicit drug abuse, violence, traffic infringement, sex crime, embezzlement, arson, murder, immigration violation, and other crimes. For each suspect, only the name of the main offense was considered, including attempts. Therefore, if suspects were arrested for violence and possession of an illicit drug, they would be placed in the violence category.

We divided psychotropic drugs into the following six groups: anxiolytics, antidepressants, sleeping pills, antipsychotics, antiepileptics, and anti‐manic drugs. Anxiolytics and sleeping pills were defined according to “Today's remedy 2020 in Japan.”^17^ The following benzodiazepines were categorized as anxiolytics: alprazolam, etizolam, oxazolam, cloxazolam, crothiazepam, dipotassium clonazepam, chlordiazepoxide, diazepam, tofisopam, fludiazepam, flutazolam, flutoprazepam, bromazepam, mexazolam, medazepam, ethyl lorazepam, and lorazepam; other benzodiazepines were categorized as sleeping pills.

### 2.3 Statistical analyses

Statistical analyses were performed using JMP^®^ version 10.0.2 (SAS Institute). The background characteristics of each group and psychotropic drug use were compared using the chi‐square test, and the odds ratios were calculated. To compare the different items of each category, the standard groups were the 20‐29 group and theft group; theft was the most common crime in Japan.

### 2.4 Ethical statement

This study was approved by the Ethical Review Committee of the Gifu University's Graduate School of Medicine on December 5, 2018 (approval no: 2018‐194). Data were collected as part of an obligatory investigation to obtain information from all suspects in the detention house but not for this study. For this reason, the opt‐out method cannot be used in a detention house. Therefore, informed consent was waived. This point was discussed and approved by the Ethical Review Committee. We were given access to the data after anonymization.

## 3 RESULTS

The background characteristics of the suspects are shown in Table 1. The data of 785 suspects were available. However, suspects with incomplete data were excluded. Therefore, 773 (680 men and 93 women) suspects were included in the study. Their average age (standard deviation) was 41.9 (15.1) years. The percentage of suspects who committed a first crime was 55.5%. The remaining 44.5% had committed multiple crimes. Theft, illicit drug abuse, violence, traffic infringement, sex crime, embezzlement, arson, murder, immigration violation, and others were the cause of detention of 38.0%, 19.1%, 7.6%, 7.4%, 4.3%, 10.6%, 1.2%, 0.9%, 5.7%, and 5.2% of the suspects, respectively.

**TABLE 1:** Background characteristics of the suspects

	Number	Percentage
Sex		
Man	680	88.0
Woman	93	12.0
Offense history		
First offense	429	55.5
Second offense	344	44.5
Age (y)		
˂20	3	0.4
20‐29	194	25.1
30‐39	183	23.7
40‐49	172	22.3
50‐59	106	13.7
60‐69	72	9.3
>70	43	5.6
Type of crime		
Theft	294	38.0
Illicit drug abuse	148	19.1
Violence	59	7.6
Traffic infringement	57	7.4
Sex crime	33	4.3
Embezzlement	82	10.6
Arson	9	1.2
Murder	7	0.9
Immigration violation	44	5.7
Others	40	5.2
Psychotropic drug		
User	205	26.5
User except for sleeping pill	129	16.7
Non‐user	568	73.5

The types of psychotropic drugs used are listed in Table 2. Of the suspects, 26.5% used all psychotropic drug types, while 16.7% used all types except sleeping pills. The most used drugs were sleeping pills (22.6%). A total of 11.1%, 3.0%, 9.6%, 1.9%, and 0.4% of the suspects used anxiolytics, antidepressants, antipsychotics, antiepileptics, and anti‐maniac drugs, respectively. Combined use of psychotropic drugs is depicted in Table 3. We found that the subjects who used antipsychotics had a tendency to combine them with other psychotropic drugs.

**TABLE 2:** Percentage of use of each psychotropic drug type

	User	Percentage
Anxiolytics	86	11.1
Antidepressants	23	3.0
Sleeping pills	175	22.6
Antipsychotics	74	9.6
Antiepileptics	15	1.9
Anti‐manic drugs	3	0.4
All psychotropic drugs	205	26.5
Psychotropic drugs, except sleeping pill	129	16.7

**TABLE 3:** Overlap of psychotropic drug use

	Anxiolytics	Antidepressants	Sleeping pills	Antipsychotics	Antiepileptics	Anti‐manic drugs
Anxiolytics	‐	15	65	37	5	2
Antidepressants	15	‐	17	13	3	1
Sleeping pills	65	17	‐	62	11	3
Antipsychotics	37	13	62	‐	10	3
Antiepileptics	5	11	3	10	‐	3
Anti‐manic drugs	2	1	3	3	3	‐

The relationship between psychotropic drug use and the crime categories is shown in Table 4. A high percentage of psychotropic drug use (38.9%) was observed among suspects for illicit drug abuse. For subjects who committed arson, this percentage was extremely high. Among suspects for violence, anxiolytics (17.0%) and antipsychotics (11.9%) were the most used. Psychotropic drug use among suspects for immigration violence (6.8%) was extremely lower than that among suspects for other crimes.

**TABLE 4:** Relationship between psychotropic drug use and crime category

	Theft	Illicit drug abuse	Violence	Traffic infringement	Sex crime	Embezzlement	Arson	Murder	Immigration violation	Others
Anxiolytics	25 (8.5%)	25 (16.9%)	10 (17.0%)	3 (5.3%)	2 (6.1%)	9 (11.0%)	2 (22.2%)	0 (0%)	3 (6.8%)	7 (17.5%)
Antidepressants	12 (4.1%)	5 (3.4%)	1 (1.7%)	0 (0%)	0 (0%)	1 (1.2%)	1 (11.1%)	1 (14.3%)	0 (0%)	2 (3.0%)
Sleeping pills	59 (20.1%)	55 (37.2%)	16 (27.1%)	8 (14.0%)	8 (24.2%)	13 (15.9%)	3 (33.3%)	1 (14.3%)	3 (6.8%)	9 (22.5%)
Antipsychotics	19 (6.5%)	26 (17.6%)	7 (11.9%)	3 (5.3%)	1 (3.0%)	6 (7.3%)	4 (44.4%)	1 (14.3%)	0 (0%)	7 (9.6%)
Antiepileptics	7 (2.4%)	3 (2.0%)	1 (1.7%)	0 (0%)	1 (3.0%)	1 (1.2%)	1 (11.1%)	0 (0%)	0 (0%)	1 (1.9%)
Anti‐manic drugs	1 (0.3%)	0 (0%)	1 (1.7%)	0 (0%)	0 (0%)	0 (0%)	0 (0%)	0 (0%)	0 (0%)	1 (2.5%)
All psychotropic drugs	69 (23.5%)	59 (39.9%)	19 (32.2%)	9 (15.8%)	9 (27.3%)	21 (25.6%)	4 (44.4%)	1 (14.3%)	3 (6.8%)	11 (27.5%)

The relationship between psychotropic drug use and crime category was investigated again, considering theft as a standard crime. The results are presented in Table 5. The findings were consistent with those outlined in Table 4. Suspects for illicit drug abuse used psychotropic drugs more than suspects for theft. Moreover, the suspects for immigration violations used psychotropic drugs less than those for theft.

**TABLE 5:** Comparison between psychotropic drug users and non‐users according to the crime category

	Psychotropic drug user		No user		Odds	
	Number	Percentage	Number	Percentage	Odds ratio	*P*
Theft	69	23.5	225	76.5	‐	‐
Illicit drug abuse	59	39.9	89	60.1	2.162	.001**
Violence	19	32.2	40	67.8	1.549	.187
Traffic infringement	9	15.8	48	84.2	0.611	.227
Sex crime	9	27.3	24	72.7	1.223	.667
Embezzlement	21	25.6	61	74.4	1.123	.664
Arson	4	44.4	5	55.6	2.609	.226
Murder	1	14.3	6	85.7	0.543	1
Immigration violation	3	6.8	41	93.2	0.239	.010*
Others	11	27.5	29	72.5	1.888	.122

* *P* < .05.

** *P* < .01.

The relationship between psychotropic drug use and the suspects’ background characteristics (eg, sex, age, and crime history) was investigated. The results are shown in Table 6. Psychotropic drug use was significantly higher among suspects in their 40s and 50s than among those in their 20s. Psychotropic drug use among female suspects was significantly higher than that among male suspects. We also found that suspects who had committed multiple crimes used psychotropic drugs more than those who had committed crimes only once.

**TABLE 6:** The relationship between the psychotropic drug use and the characteristics of subjects

	Psychotropic drug user	No user	Odds	*P*
Age (y)				
<19	1	2	2.122	.476
20‐29	37	157	‐	‐
30‐39	48	135	1.509	.109
40‐49	60	112	2.273	.001^**^
50‐59	40	66	2.572	.001^**^
60‐69	11	61	0.765	.591
>70	8	35	0.97	1
Sex				
Male	171	509	‐	‐
Female	34	59	1.715	.024^*^
Offense history				
−	87	342	‐	‐
+	118	226	2.052	.000^**^

* *P* < .05.

** *P* < .01.

## 4 DISCUSSION

This is the first survey to analyze the relationship between psychotropic drug use and crime categories in Japan and other countries. We found that 26.5% of suspects used psychotropic drugs. When users of sleeping pills were excluded, the psychotropic drug use rate was 16.7%. Mishima et al^18^ studied psychotropic drug use in the general population of Japan. According to their study, the use rates of sleeping pills, anxiolytics, antidepressants, and antipsychotics were 5.00%, 4.72%, 2.62%, and 0.89%, respectively. The use rates of sleeping pills, anxiolytics, antidepressants, and antipsychotics were 22.6%, 11.1%, 3.0%, and 9.6%, respectively, in our survey. Therefore, the use rate of psychotropic drugs except antidepressants was much higher among suspects than in the general population.

We found that psychotropic drug use was significantly higher among suspects in their 40s and 50s than those in their 20s. Psychotropic drug use increases with aging. However, the use rates in suspects in their 60s and 70s were not high. The reason for this is unknown. In addition, psychotropic drug use was higher among female than male suspects. Some studies reported that the prevalence of mental illnesses was higher in women than in men.^15^, ^16^ Thus, Japan may have similar tendencies with other countries. Although we hypothesized that the high prevalence of mental illnesses or high psychotropic drug use was caused by the high percentage of illicit drug abuse among female suspects, the percentages of illicit drug users among all suspects were 21.3% for men and 21.5% for women. This finding ruled out our hypothesis. Contrarily, Mishima et al reported that psychotropic drug use was slightly higher in women in the general population.^18^ This may explain our findings. In this study, we also found that suspects with a multiple crime history used psychotropic drugs more frequently than those who had committed crimes only once. There is no similar previous study. Mental illness is more common among suspects with a multiple crime history. Based on this, appropriate mental health services are required for these suspects to prevent further crimes. This could be the objective of future research.

Our answer to the research question was that 26.5% of the residents in the detention house used psychotropic drugs. The psychotropic drug use rate (excluding sleeping pill use) was 16.7%. The latter percentage is almost equal to the prevalence of mental illnesses in prisons.^11^ However, the White Paper on Crime 2019 from the Japanese Ministry of Justice reported that the percentage of crimes committed by people with mental disabilities was only 1.0%.^10^ We consider that this large discrepancy in the prevalence of mental illnesses should be considered during judgment, and being imprisoned might lead to the negligence of mental illness in the process of judgment. This percentage is higher than that in the general population of Japan. This result might show the importance of the system which we can diagnose suspects and attribute to an appropriate treatment process for them. Psychotropic drug use was high among suspects for illicit drug use and low among suspects for immigration violence.

This study has some limitations. First, we were unable to obtain the suspects’ diagnostic information; we could obtain only information on psychotropic drug use. Second, the quantitative information on drug use was incomplete and was not reliable. However, this is the first survey to report the psychotropic drug use rates and the relationship between psychotropic drug use and crime categories in a detention house. Therefore, our findings are valuable, despite the limitations.

## CONFLICT OF INTEREST

The authors declare no conflict of interest.

## AUTHOR CONTRIBUTION

All the processes of writing this article were done by AN.

## ETHICAL APPROVAL

This study was approved by the Ethical Review Committee of the Gifu University's Graduate School of Medicine on December 5, 2018 (approval no: 2018‐194). Data were collected as part of an obligatory investigation to obtain information from all suspects in the detention house but not for this study. For this reason, the opt‐out method cannot be used in a detention house.

## INFORMED CONSENT

For the above reasons, informed consent was waived. This point was discussed and approved by the Ethical Review Committee. We were given access to the data after anonymization.

## Data Availability

The data that support the findings of this study are available on request from the corresponding author. The detention house which provided the data to us understood the value of our research and admitted only our research. Therefore, if someone wants to use the data, we will try to get an approval from the detention house. We hope your understanding of the particular position of the detention house.
